# Assessment of functioning in Dutch primary care: Development study of a consultation tool for patients with chronic conditions and multimorbidity

**DOI:** 10.1111/hex.13474

**Published:** 2022-05-24

**Authors:** Simone Postma, Henk Schers, Tom van de Belt, Kees van Boven, Huib ten Napel, Hugo Stappers, Debby Gerritsen, Tim Olde Hartman

**Affiliations:** ^1^ Department of Primary and Community Care Radboud University Medical Center Nijmegen The Netherlands; ^2^ WHO Collaborating Centre for Family of International Classifications Nijmegen The Netherlands; ^3^ Radboud REshape Innovation Center Radboud University Medical Center Nijmegen The Netherlands

**Keywords:** consultation tool, design thinking, disability and health, International Classification of Functioning, multimorbidity, participatory development process, primary care

## Abstract

**Background:**

In primary care, a shift from a disease‐oriented approach for patients with multimorbidity towards a more person‐centred approach is needed.

**Aim:**

To transform a self‐report questionnaire for patients with chronic conditions in primary care, the Primary Care Functioning Scale (PCFS), into an understandable, visually attractive and feasible consultation tool for patients and health care providers. The consultation tool consists of a web‐based version of the PCFS, which is filled in by the patient and is processed to a feedback report that summarizes and visualizes the main findings. The feedback report can be discussed with the patient to facilitate a more person‐centred conversation for patients with chronic conditions and multimorbidity in general practice.

**Design and Setting:**

In this qualitative study, we developed the consultation tool by using design thinking in a participatory developmental process.

**Methods:**

In the first phase, we constructed five different feedback report templates to summarize and display the results of a completed PCFS questionnaire in a series of two expert meetings with patients and general practitioners (GPs). In the second phase, we performed an exploratory qualitative interview study involving dyads of patients with chronic conditions and their practice nurses. In an iterative process, we explored their experiences with the consultation tool.

**Results:**

Patients, as well as GPs, preferred a clear manner of presenting the results of the questionnaire in a feedback report. In 18 interviews with patients and practice nurses during three different interview rounds, we adjusted the feedback report and consultation tool based on the input from patients and practice nurses. After the final interview round, patients and practice nurses consented that the consultation tool was useful for having a more in‐depth consultation about functioning and patients' preferences when integrated into the regularly scheduled consultations.

**Conclusion:**

We were able to develop an understandable and feasible consultation tool that is applicable in already existing chronic disease management programmes in general practice in the Netherlands.

**Patient or Public Contribution:**

To increase the understandability and feasibility of the consultation tool, we collaborated with end‐users and actively involved patients, GPs and practice nurses in a participatory development process.

## 1 INTRODUCTION

The number of patients with chronic morbidity and multimorbidity in general practice is still increasing.^1^ This poses a major challenge for health care providers, such as general practitioners (GPs) and practice nurses. In the Netherlands, almost all citizens are listed in one general practice. The care for chronic conditions, such as diabetes mellitus (DM), cardiovascular diseases (CVDs) and chronic obstructive pulmonary disease (COPD) is generally organized in disease management programmes operated by GP co‐operatives. Patient care itself for these conditions is provided by practice nurses in their own GP practice, supervised by GPs. Management and follow‐up are based on the practice guidelines of the Dutch College of General Practitioners. Guidelines for patients with chronic morbidity are elaborately focusing on one disease and disease‐related outcome measures.^2^ However, focusing on a single condition may lead to less attention for the impact of having multiple chronic conditions on the level of daily functioning.^3^, ^4^ Patients with multimorbidity report reduced levels of daily functioning, physical well‐being and quality of life, and have worse health outcomes compared to patients without chronic morbidities.^5^, ^6^, ^7^, ^8^ Patients with multiple chronic conditions also experience barriers to self‐care, for instance, engaging in activities promoting physical and psychological health and managing the impact of the illness on physical, psychological and social functioning.^9^, ^10^, ^11^ A disease‐oriented approach for patients with multiple chronic conditions in general practice, therefore, has serious limitations, and a more person‐ and context‐centred approach that focuses on the consequences of chronic conditions and multimorbidity in daily life is needed. However, professionals in primary care are hardly equipped nor trained to explore patients' levels of functioning and preferences.

Recently, we developed a self‐report questionnaire that assesses the different aspects of functioning and satisfaction in patients with chronic conditions in primary care; the Primary Care Functioning Scale (PCFS).^12^ The PCFS is based on the International Classification of Functioning, Disability and Health. It can be used as a valid and reliable questionnaire for measuring the level of functioning in individual patients of 50 years or older with multiple chronic conditions.^13^ The PCFS might also be a suitable tool to enhance a discussion about the patient's level of functioning during consultations and might help the practice nurse and GP to adopt a more person‐ and context‐centred approach in which the consequences of multiple chronic conditions are addressed.

The aim of this study was to develop a consultation tool out of the PCFS questionnaire for general practice. The consultation tool consists of a web‐based version of the PCFS, which is filled in by the patient and is processed to a feedback report that summarizes and visualizes the main findings. The feedback report can be discussed with the patient to facilitate a more person‐centred conversation for patients with chronic conditions and multimorbidity in general practice.

## 2 METHODS

### 2.1 Study design and setting

The consultation tool was developed by using the principles of human‐centred design; that is, a participatory development process, with the active involvement of patients, GPs and practice nurses.^14^, ^15^, ^16^, ^17^ The human‐centred design approach is used in healthcare to develop services, physical spaces and processes that meet the needs of health care providers and patients. It involves the perspectives of end‐users and includes methods, such as brainstorming and discussions, identifying user needs and collaboration. Typically, human‐centred design follows the principles, such as empathize, define, ideate, prototype and test in subsequent phases.^18^ To transform a completed PCFS questionnaire into a consultation tool that can be used in daily consultations between the patient and the health care provider, we formulated the following requisites for the consultation tool: (1) the PCFS questionnaire can be completed by patients at home and automatically generates a feedback report based on the results, (2) this feedback report is feasible for use by patients and their health care provider during a consultation to discuss a patient's functioning. The consultation tool was developed in two phases. At the beginning of the study, we aimed to develop the consultation tool for patients and health care providers in general practice, primarily GPs. However, during the process, it became clear that the usage of this consultation tool suited the practice nurse more than the GP.

In the first phase (A), patients, GPs and researchers developed different feedback report templates that provide a summary of the results of the PCFS questionnaire. In the second phase (B), these feedback report templates were tested in consultations among patients and practice nurses in an iterative interview study.

### 2.2 Participants

For the development of the feedback report template in Phase A, six patients with chronic conditions included in chronic disease management programmes in their general practice were invited to participate in two expert panel meetings. These patients were selected by their GP and we asked the GPs to maximize the variance in age, education, life experience and estimated level of functioning of their patients. The patients were age 50 years or older with the presence of DM 1 or 2, COPD/asthma or a CVD and already familiar with the PCFS from a previous validation study. They consented at that time that they may be contacted for follow‐up studies.^12^ Next to the six patients, the expert panel consisted of their three GPs and three researchers, representing both patient and professional expertize in chronic conditions.

For the interview study among patients and practice nurses in Phase B, we recruited 18 participants (nine dyads of a patient with multiple chronic conditions and their practice nurse) from seven different primary care practices located in urban and rural areas within the Radboudumc Practice‐Based Research Network, The Netherlands. Patients had to participate in chronic disease management programmes for DM, COPD/asthma or CVD.

Nine practice nurses each randomly invited one patient with DM, COPD/asthma or CVD above the age of 50 years to participate in this study. The study was carried out according to Dutch legislation and the declaration of Helsinki. The accredited Medical Research Ethics Committee Radboudumc Nijmegen gave clearance to conduct the study (registration number 2017‐3936).

From every patient and practice nurse, informed consent was obtained; patients and practice nurses were able to withdraw their consent at any time.

### 2.3 Development of feedback report templates

**Figure A1:**
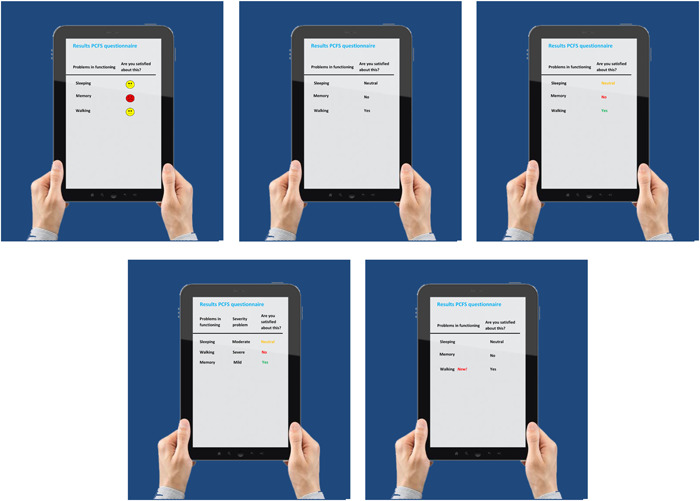
Five different feedback report templates

In a series of two subsequent expert meetings, patients and GPs collaborated within an expert panel and discussed key features of the results of the questionnaire that were deemed relevant for the feedback report template. To identify the feedback report's user needs, patients and GPs were asked which results from the questionnaire were important to report for the patient and the professional. Also, they were asked to illustrate in drawings using colours, figures, tables, graphics and emoticons, how the results of the questionnaire are preferably reported. After the second expert meeting, on the basis of the discussions, five different feedback report templates could be constructed for use in the second phase of this study (Figure A1).

### 2.4 Testing with patients and practice nurses

Patients participating in chronic disease management programmes were asked by their practice nurse to complete the PCFS questionnaire at home. Next, patients discussed the results of the questionnaire and feedback report templates within 2 weeks in a 20‐min consultation with the practice nurse. After the consultation, patients and practice nurses were interviewed separately about the experiences with the use of the questionnaire, feedback report templates and the consultation.

#### 2.4.1 Interview procedure

An interview guide was developed for the interviews with the patients and practice nurses based on the Consolidated Framework for Implementation Research (CFIR; see Table 1 for topics). In addition, the different feedback report templates were demonstrated to the patients and practice nurses during the interviews. All face‐to‐face interviews were conducted at the patient's home and practice nurse's practice. Interviews lasted 30–60 min and were conducted by two researchers; one researcher conducted the interviews in the first round (F. H.; medical student) and a second researcher conducted the interviews in the following interview rounds (S. P.; GP and PhD student). All interviews were recorded on audiotape and transcribed verbatim afterwards.

**Table 1:** Interview guide topic box

Patient	Practice nurse
Questionnaire	Questionnaire
*How did you experience the questionnaire in general?*	*How did you experience the questionnaire in general?*
*What do you think about the relevance of the questions?*	*What do you think about the relevance of the questions?*
*Do the questions provide more insight in your daily functioning?*	*Do the questions provide more insight in your patient's daily function?*
*Is the questionnaire relevant for your practice nurse and in which way?*	*Is the questionnaire relevant for you as a professional and in which way?*
*Can you provide suggestions for improving the questionnaire?*	*Can you provide suggestions for improving the questionnaire?*
Presentation of the feedback report template	Presentation of the feedback report template
*What do you think about template 1–5?*	*What do you think about template 1–5?*
*Do you prefer any template and why?*	*Do you prefer any template and why?*
*Do you have any suggestions that could improve the design of the template?*	*Do you have any suggestions that could improve the design of the template?*
*Do you think that the practice nurse and patient should receive the same feedback report?*	*Do you think that the practice nurse and patient should receive the same feedback report?*
Applicability questionnaire and feedback report	Applicability questionnaire and feedback report
*How did you prepare the consultation?*	*How did you organize the consultation?*
*How did you experience a consultation that focuses on your own functioning?*	*How did you experience a consultation that focuses on the functioning of your patient?*
*Did the feedback report and/or consultation about functioning motivate you to improve your functioning in daily life and how?*	*Did the feedback report and/or consultation about functioning affect the way you provide care to your patient and how?*
*When should the results of the questionnaire be discussed?*	*When should the results of the questionnaire be discussed?*
*Would you like to have a training or instruction in how the consultation tool can be used?*	*Would you like to have a training or instruction in how the consultation tool can be used?*
*When do you want to receive the results of the questionnaire?*	*When do you want to receive the results of the questionnaire?*
*How much time should there be between the completion of the questionnaire and the consultation with the practice nurse?*	*How much time should there be between the completion of the questionnaire and the consultation with the practice nurse?*
*In which way should the consultation tool be integrated in the periodic checks of a patient with a chronic condition?*	*In which way should the consultation tool be integrated in the periodic checks of a patient with a chronic condition?*
*How do you see the role of the practice nurse in relation to the consultation tool?*	*How do you see the role of the practice nurse in relation to the consultation tool?*

#### 2.4.2 Analysis of the interviews

The interviews of the first round were analysed by using constant comparison analysis, an iterative process of coding, analysis and discussion.^19^ After the first round of interviews, two researchers (F. H. and S. P.) independently read all transcripts several times to familiarize themselves with the data. Each analysis was compared and discussed in a consensus meeting. After the interviews from round 1, two researchers defined categories independently and these were discussed with a third researcher (T. o. H.; GP and PhD). The developing categories were constantly matched with the transcripts and saturation was reached until no new categories were found during this coding process. Different themes in patients' and practice nurses' experiences emerged from this process of coding, analysis and discussion (constant comparative analysis).^19^ For the second round of interviews, two researchers (S. P., K. v. B.; GP and PhD) analysed the data from the transcripts independently based on the identified themes in the first round of interviews. Revisions were made in the feedback report template and the structure of the consultation between the patient and the practice nurse, based on the emerged difficulties, problems and suggestions from the preceding analysis in a consensus procedure by three researchers (S. P., K. v. B., T. o. H.). As we analysed the data in an iterative process, the process of data collection continued until no more new difficulties, problems, and suggestions were mentioned by practice nurses or patients.

## 3 RESULTS

### 3.1 Development of feedback report templates

Patients and GPs revealed the following results from the questionnaire as important and relevant to include in the feedback report according to the patient and GP: (1) answers scored with a problem and (2) answers scored with dissatisfaction. Most patients and GPs preferred that the results of the PCFS were reported in written text (vs. the use of images and figures). Regarding the content of the presentation in the feedback report of the results, there was a variety of opinions among patients and GPs about (1) whether the level of the problem should be reported (from a mild problem to severe problem), (2) the need for a sum score of the items, (3) the use of colour in expressing the level of problem and satisfaction, (4) the use of symbols to illustrate the item, (5) the use of symbols with a facial expression (i.e., emoji) to illustrate the level of satisfaction and (6) whether to display changes over time in the level of functioning. After the second expert meeting, based on the input from patients and GPs, five different feedback report templates were constructed to use for further development in the subsequent testing with patients and practice nurses (Figure A1).

### 3.2 Testing with patients and practice nurses

In total 18 participants (nine dyads of one patient and one practice nurse) consented to participate. They were interviewed in the period from October 2018 to March 2020. The participants' characteristics in relation to the chronic disease management programme are presented in Table 2. In the first round of interviews, three dyads participated; in the second round of interviews, three other dyads participated, and in the third round a further two dyads participated.

**Table 2:** Participants' characteristics

Couple	Patient	Practice nurse
	*Participation chronic disease management programme(s) (age in years, gender)*	*Expertize*
1	COPD (62, female)	DM, COPD/asthma (female)
2	DM (71, female)	DM, COPD/asthma, and CVD (female)
3	DM (78, female)	DM, COPD/asthma, and CVD (female)
4	DM and CVD (62, female)	DM, COPD/asthma, and CVD (female)
5	DM and COPD (73, male)	DM, COPD/asthma, and CVD (female)
6	DM (53, female)	DM, COPD/asthma, and CVD (female)
7	DM (70, female)	DM, COPD/asthma, and CVD (female)
8	DM and CVD (68, female)	DM, COPD/asthma, and CVD (female)
9	CVD (59, male)	CVD (female)

Abbreviations: COPD, chronic obstructive pulmonary disease; CVD, cardiovascular disease; DM, diabetes mellitus.

We could identify four relevant main themes of experiences with the consultation tool in general practice: (1) content of the questionnaire, (2) feedback report template (i.e., presentation of the results), (3) communication about functioning during a consultation and (4) applicability of the consultation tool in daily practice. In the following section, the results of the different interview rounds will be presented per theme and which identified difficulties, problems and suggestions resulted in a revision of the consultation tool after an interview round.

#### 3.2.1 Content questionnaire

All patients and practice nurses were positive about the content of the PCFS questionnaire consistently through every interview round. Also, all practice nurses indicated that the PCFS is a complete, extensive and broad questionnaire capturing the relevant aspects of functioning. The questionnaire provided the practice nurses with more knowledge about their patients' daily functioning, and this was regarded as an additional value in the care for their patients.

*Extensive. So, clearly quite different areas: psychosocial, emotional and physical too. That diversity was very clear in the questionnaire. Well, it really gives you more information about the patient. I've actually known this patient for years, but I really learned new things […]*

*Of course, when you have a short consultation you always discuss a small, specific subarea, mainly to do with the disease and the disorders. And now I have a bit of a better picture of ‘hey, who is she exactly? What does she do in her spare time?’ So it helped me get to know her a bit better*. (Nurse practitioner: female, expertize DM, COPD/asthma—Round 1)

All patients indicated that the PCFS provided a good and complete impression of the different aspects of their own functioning and that the items of the questionnaire captured important, personal and relevant information for the health care professionals about themselves.

#### 3.2.2 Feedback report templates

The analyses of the first six interviews with patients and practice nurses demonstrated important difficulties and problems with the interpretation of the different feedback report templates. For example, one template illustrated problems in functioning in the first column and satisfaction levels with emoticons in the second column (Figure A1). Patients and practice nurses interpreted the level of satisfaction as the level of the problem in functioning differently than the researchers intended. For example, the template illustrated there was a problem in sleeping in the first column and the second column indicated that there was no dissatisfaction about the experienced problem in sleeping. Most practice nurses and patients interpreted that there was no problem with sleeping.

*Right, then I start to get doubts because I thought ‘I sleep well, so that's the smiley’ but that's* apparently *not the function. So that might get a bit confusing*. (Patient: female, 78 years—Round 1)

Also, patients and practice nurses found the addition of the use of symbols in the templates confusing, or difficult to understand what the symbol was representing or of no added value.

*The* younger *generation will like that symbol smiley with a ‘zzz’ (sleeping symbol). An elderly, or my father, would not understand what it means*. (Patient: female, 62 years—Round 1)

Based on the emerging difficulties and problems with the understandability of the feedback report templates in all of the first interviews with patients and practices nurses, we constructed a new feedback report template that included only the basic requirements mentioned by patients and GPs in the preceding development of the feedback templates: A written feedback report, including items scored with a problem and/or items scored as dissatisfaction, in addition to the levels of the problems in functioning and satisfaction. After the adjustments made in the feedback report, no more difficulties and problems nor new suggestions were mentioned by patients and practice nurses in the following interview rounds regarding the understandability of the feedback report or preferences in the layout of the feedback report.

#### 3.2.3 Communication about functioning during a consultation

Patients experienced that the consultation focused on the various aspects of functioning and that this differed from the regular follow‐up consultations. They regarded this as a positive change of the consultation and mentioned the consultation as being pleasant, personal and relevant.

*It was much more personal. We got to talk about things that I've often thought I'd like to say something* about *but didn't come up in the check‐up because you just got those ‘yes’, ‘no’, ‘yes’, ‘no’ questions, right? […] For example, I had this pain in my body and I'd mentioned it a couple of times but then you got the next question so you never got an answer to it*. (Patient: female, 53 years—Round 2)

Practice nurses considered the results of the PCFS as a useful tool to reach a more in‐depth consultation about functioning. In general, practice nurses found that the consultation provided more information about the different aspects of a patient's functioning, also for practice nurses with a long‐term nurse–patient relationship.

*You can give* people *this very clear feeling of ‘I see you, you haven't come here because…, I see what you're struggling with, what's important to you’. […] I think that could be really nice for people. […] That they definitely feel someone is listening and that they say that too*. (Nurse practitioner: female, expertize DM, COPD/asthma and CVD—Round 2)

#### 3.2.4 Applicability of the consultation tool in the daily practice

During the first round of interviews, practice nurses indicated that they found it difficult to structure the consultation and it was not clear what needed to be discussed exactly during the consultation. The consultation lengths exceeded the suggested duration of 20 min because all the problems and dissatisfactions were discussed, and in general the practice nurses were missing clear instruction.

*So I do think it is important for the nurse practitioner to get some instructions in advance. […] But it did cost me quite a lot of time. Twenty minutes? With these questions—so many? I definitely can't manage that. [Laughs]*. (Nurse practitioner: female, expertize DM, COPD/Asthma and CVD—Round 1)

After round 1 of the interviews, based on the experiences and suggestions from the practice nurses, clear instructions for the consultation for the practice nurses were created in which the consultation should clarify the patient's: (1) current problems, (2) needs (i.e., satisfaction levels) and (3) preferences to change certain specific limitations of functioning and help to set achievable goals with defined actions necessary to reach these goals. After the second round of interviews, practice nurses were satisfied with the instructions that helped them to structure their consultation with the patient in approximately 20–30 min. In relation to the instructions, after interview round 2, it was suggested that the consultation should focus on one or two problems in functioning from the feedback report that is chosen in a consensus between the patient and the practice nurse because of the limited time of the consultation to discuss all problems. After the new adaptations of the practice nurses' instructions, no new problems and suggestions were mentioned in the third round of interviews.

Practice nurses indicated that the conversation about functioning should be integrated with the existing regular follow‐up consultations of the chronic disease management programmes to be able to stimulate improvement in certain aspects of functioning.

*I would link it to the regular consultations. […] yes, with some diabetes patients I reckon you can say, hey, I see this, or I think that's related to the diabetes or even a consequence of it, so let's talk about that next time*. (Nurse practitioner: female, expertize DM, COPD/Asthma and CVD—Round 2)

Patients also argued that an integration of the consultation with the existing regular follow‐up for the chronic disease is preferred because of better time management.

*Then you have indeed covered everything at that point, otherwise you have to come back for something* else *in two weeks' time, let's say, and I can imagine that being a problem, people saying ‘what, do I have to come* again*?’ […] I would indeed prefer… right, that it should fit in with that overall… right, so you say, now I know where I'm at*. (Patient: male, 73 years—Round 2)

In the third round, the consultation about functioning was integrated into the existing follow‐up consultation for the chronic disease. Patients indicated that it felt in place to discuss problems and difficulties in their daily functioning in combination with their regular consultation with the practice nurse and no new problems, difficulties or suggestions were mentioned by patients. Also, the instruction to integrate the consultation with the scheduled chronic disease management consultation was clear for practice nurses. A practice nurse from the last interview round indicated that she experienced the combination of the consultation tool and the annual check as ‘normal’.

*I basically just went through the annual check‐up calmly and at the start I began with ‘right, you filled in the questionnaire and that showed two highlights, right?’ and then I basically incorporated those two points in the conversation. And I found the annual check‐up didn't feel that different at all to normal. I had the feeling that we covered a lot of ground but* those *points that he'd suggested, you could delve into them more specifically. I found the explanation I read in advance very clear, the instructions were clear, I really liked that summary, it was very workable, those results. *(Nurse practitioner: female, expertize CVD—Round 3)

## 4 DISCUSSION

### 4.1 Summary

In this study, we developed a consultation tool to assess and discuss the functioning of patients with chronic morbidity in the general practice. Through different steps according to the participatory developmental process and by using design thinking we transformed the PCFS questionnaire into an understandable, visually attractive and feasible consultation tool for patients and health care providers. Patients and practice nurses were positive about the added value of a consultation that focuses on the functioning and should be integrated with the regular follow‐up consultations of the chronic disease management programmes in daily practice.

### 4.2 Comparison with the literature

A number of studies to date have explored the effectiveness of interventions that aim to support the management of multimorbidity in primary care for improvement in health outcomes.^20^ However, results do suggest that more emphasis should be given to the empirical analysis of the impact of multimorbidity on a person's functioning.^20^, ^21^

It is known that health‐related self‐report questionnaires can motivate patients to reflect on their own health and help to discuss these issues with their clinicians ‘in their own words’, which supports the communication process and can improve health care outcomes.^22^, ^23^, ^24^, ^25^

In this study, we have demonstrated that the results of the PCFS questionnaire can be used as a consultation tool by the patient and the health care provider incorporating the consequences of chronic conditions and multimorbidity on daily life functioning, thereby stimulating personalized healthcare. Practice nurses indicated that they found it useful to connect the problems raised by the PCFS questionnaire with the patients' known chronic diseases and this was an important added value of the consultation tool to their regular care.

The involvement of patients and/or end‐users in a participatory process is widely accepted and is known to be important for developing a successful intervention.^26^, ^27^, ^28^, ^29^ In another study developing an eHealth tool for patients with complex chronic disease and disability, qualitative research methods were incorporated into a user‐centred design, leading to a major shift in the purpose and design of the prototype tool.^29^ This was also the case in our study in which the consultation tool was adapted after every interview round to better suit the needs of the patients and practice nurses in daily practice, for example, most of the developed illustrations with the use of colours, figures, tables, graphics and emoticons in the feedback report were not preferred by patients and practice nurses.

Practice nurses indicated that an important added value of the consultation tool is the connection of the raised problems in functioning with the patients' chronic diseases. However, practice nurses, as well as patients, doubted whether the consultation tool would have this added value when it would be used apart from the existing regular follow‐up consultation.

### 4.3 Strengths and limitations

The most important strength of this study is that the consultation tool was developed in collaboration with all stakeholders involved, such as patients, GPs and practice nurses. This is in line with the development and validation of the PCFS in which patients were also actively involved.^12^, ^13^

Next to the participatory development process, we used the CFIR framework for the qualitative interviews. The CFIR provides a framework of constructs to increase effective implementation of interventions in healthcare that can be used as a practical guide for systematically assessing the potential barriers and facilitators to overcome potential barriers for implementation at a later stage.^30^ A limitation of this study is that there might be a slight bias in the recruitment of the patients. Although not intended, female participants were overrepresented in the second part of the study. This may have resulted in differences in individual experiences; however, it is unlikely that this has influenced the acceptability and feasibility of the consultation tool.

### 4.4 Implications for research and/or clinical practice

**Figure A2:**
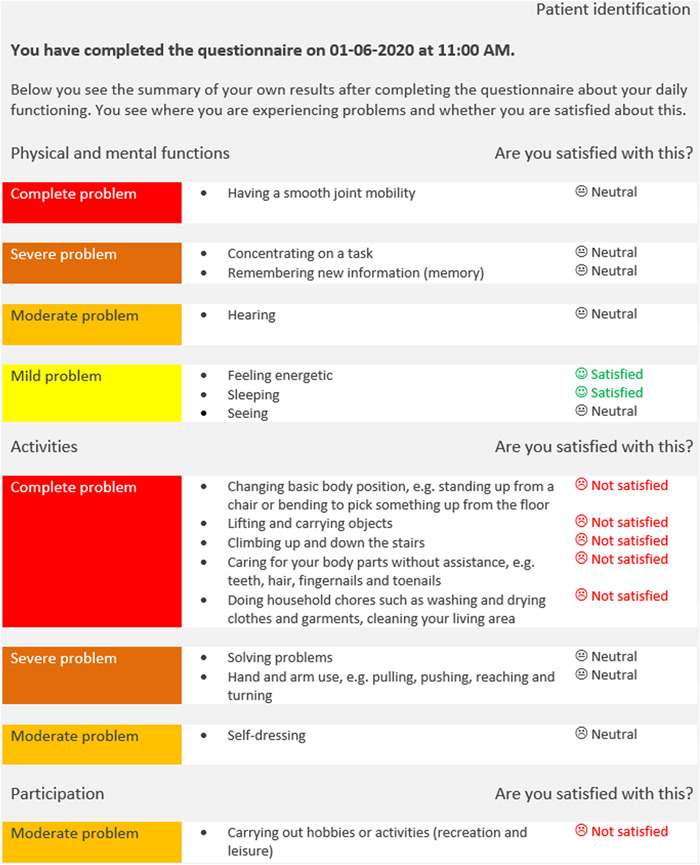
Definite version of the consultation tool

Alongside this study, we refined the consultation tool, which now automatically generates a personalized feedback report based on the patient's self‐reported answers to the questionnaire. The final version of the feedback report is presented in Figure A2. In the following study, we will test the further feasibility and potential effectiveness of the consultation tool in a randomized control trial in general practice. To answer further questions about the implications of this consultation tool and whether it can be used for managing chronic conditions and multimorbidity more widely, we also need to validate and test the consultation tool for potential effectiveness in other patient populations outside chronic management programmes within the Dutch health care context.

## 5 CONCLUSION

With the involvement of the stakeholders, we developed an understandable, visually attractive and feasible user‐centred consultation tool for patients and health care providers, which supports the integration of the different aspects of health‐related functioning in existing chronic disease management programmes. The consultation tool can be used in the general practice to shift the regular follow‐up consultations for patients with chronic conditions, that is still mainly focused on single disease outcomes measures, towards a more person‐centred consultation taking the consequences of chronic suffering on health‐related functioning into account.

## AUTHOR CONTRIBUTIONS

*Conceived and designed the analysis*: Simone Postma, Henk Schers, Tom van de Belt, Kees van Boven, Huib ten Napel, Hugo Stappers, Debby Gerritsen and Tim olde Hartman. *Collected the data*: Simone Postma, Henk Schers, Tom van de Belt, Kees van Boven, Huib ten Napel, Fedor Hagema and Tim olde Hartman. *Contributed data or analysis tools and performed the analysis*: Simone Postma, Henk Schers, Tom van de Belt, Kees van Boven, Huib ten Napel and Tim olde Hartman. *Wrote the paper*: Simone Postma, Henk Schers, Tom van de Belt, Kees van Boven, Huib ten Napel, Hugo Stappers, Debby Gerritsen and Tim olde Hartman.

## CONFLICTS OF INTEREST

The authors declare that there are no conflicts of interest.

## Data Availability

The data that support the findings of this study are available from the corresponding author upon reasonable request.

## APPENDIX A

See Figures A1 and A2
